# Description of a new species of *Metabemisia* Takahashi, 1963 from China (Hemiptera, Aleyrodidae)

**DOI:** 10.3897/zookeys.604.8203

**Published:** 2016-07-11

**Authors:** Ji-Rui Wang, Yu-Zhou Du

**Affiliations:** 1 School of Horticulture and Plant Protection & Institute of Applied Entomology, Yangzhou University, Yangzhou 225009, China; 2 School of Agricultural & Food Science, Zhejiang Agriculture & Forestry University, Linan, 311300, China

**Keywords:** Aleyrodidae, Metabemisia, taxonomy, new taxa, China

## Abstract

A new whitefly species, *Metabemisia leguminosa* **sp. n.**, collected from an undetermined leguminous herb is described from Wuzhishan Mountain, Hainan Island, China. The puparium of the new species differs from that of all other *Metabemisia* species by the presence of 4–5 rows of very small distinct papillae along the margin, the absence of the first abdominal seta, and the indistinct thoracic tracheal pores. An identification key to the worldwide species of *Metabemisia* is provided.

## Introduction

The genus *Metabemisia* (Hemiptera: Aleyrodidae) was established by Takahashi (1963) with *Metabemisia distylii* Takahashi as its type species by monotypy. Only three species have hitherto been placed in this genus. Takahashi (1963) described *Metabemisia distylii* from Japan on *Distylium racemosum*. Mound (1967) described *Metabemisia filicis* from Scotland on *Dryopteris* sp., *Nephrolepis* sp. and *Davallia* sp.; from England on *Pteris togoensis* or *Cyclosorus dentate*, and Ko et al. (1998) recorded the same species from Taiwan (China) on *Tectaria decurrens*. Martin (2001) described *Metabemisia palawana* from Philippines on *Lastreopsis* sp.

A faunal survey of Aleyrodidae was conducted in some nature reserves of Hainan Island in May 2012 as the Aleyrodidae fauna in these areas had not been previously investigated in detail. Puparia of an undescribed species of this genus were collected from Wuzhishan Mountain, this being the first record of the genus from Mainland China.

## Material and methods

The puparia of the new species were collected from an undetermined leguminous herb found on Wuzhishan Mountain, 18°51'N, 109°39'E, 561 m, Hainan Island, China on 18 May, 2012. The puparia were mounted on glass slides following the method suggested by Martin (1987), as compared with other methodologies such as the method given by Hodgesa and Evans (2005) and Dubey and David (2012), the steps are almost same except slight differences. The terminology for morphological structures follows Bink-Moenen (1983), Martin (1985) and Gill (1990). The measurements were made through measuring 9 specimens including the holotype, using a LEICA MZAPO stereo-microscope. The Scanning Electron Microscope (SEM) images were taken using Philips XL30-Environmental Scanning Electron Microscope at 20 kV/EHT and 80 Pa between 157 to 1258 times magnification. The detail steps of SEM study following Wang et al. (2014).

The holotype is deposited in the Insect Collection of Yangzhou University
(YZU). A paratype will be deposited in each of the following institutions: Natural History Museum
(BMNH), London, UK; Zoological Survey of India
(ZSI), Kolkata, India; the remainder of the paratypes are currently deposited in Insect Collection of Yangzhou University and Shanghai Entomological Museum, Chinese Academy of Sciences
(SHEM).

## Taxonomy

### Metabemisia

Taxon classificationAnimaliaHemipteraAleyrodidae

Takahashi

Metabemisia Takahashi, 1963: 52. Type species: Metabemisia distylii, by monotypy.

#### Diagnosis.

Puparium elliptical, with a single row of submarginal setae, *Metabemisia distylii* and *Metabemisia filicis* bear ten pairs of submarginal setae while *Metabemisia palawana* bears 14 pairs. Vasiform orifice elongate-cordate to triangular, much longer than wide, the trapezoidal operculum occupying about half of orifice (Takahashi 1963; Martin and Camus 2001). This genus resembles *Parabemisia* Takahashi in the shape of puparium and the presence of a row of submarginal setae, but can be distinguished by the lingula wanting lateral tubercles and in the presence of caudal tracheal cleft. It also resembles *Neomaskellia* Quaintance & Baker, but differ in the characters of vasiform orifice.

### Metabemisia leguminosa sp. n.

Taxon classificationAnimaliaHemipteraAleyrodidae

http://zoobank.org/CAFBAA3C-E0BA-45D5-8BD7-90DE08FB377E

Figs 1–4 , 5–9 , 10–11 , 12

#### Type locality.

China, Hainan Island, Wuzhishan Mountain, 18°51'N, 109°39'E, 561 m, on Leguminous herb, 18.v.2012, leg. JR Wang.

#### Type material.

Holotype: China, Hainan Island, Wuzhishan Mountain, 18°51'N, 109°39'E, 561 m, 1 puparium on slide, on leguminous herb, 18.v.2012, leg. J R Wang (WZS-NO.1), deposited in YZU.

Paratypes: Fifteen paratypes, same data as the holotype, 15 puparia on 15 slides, (WZS-NO.2–4: BMNH-1, ZSI-2); (WZS-NO.5–16: SHEM-2, YZU-10). 17 dry puparia on leguminous leaves with above collection data available at YZU.

#### Diagnosis.

**Figures 1–4.**
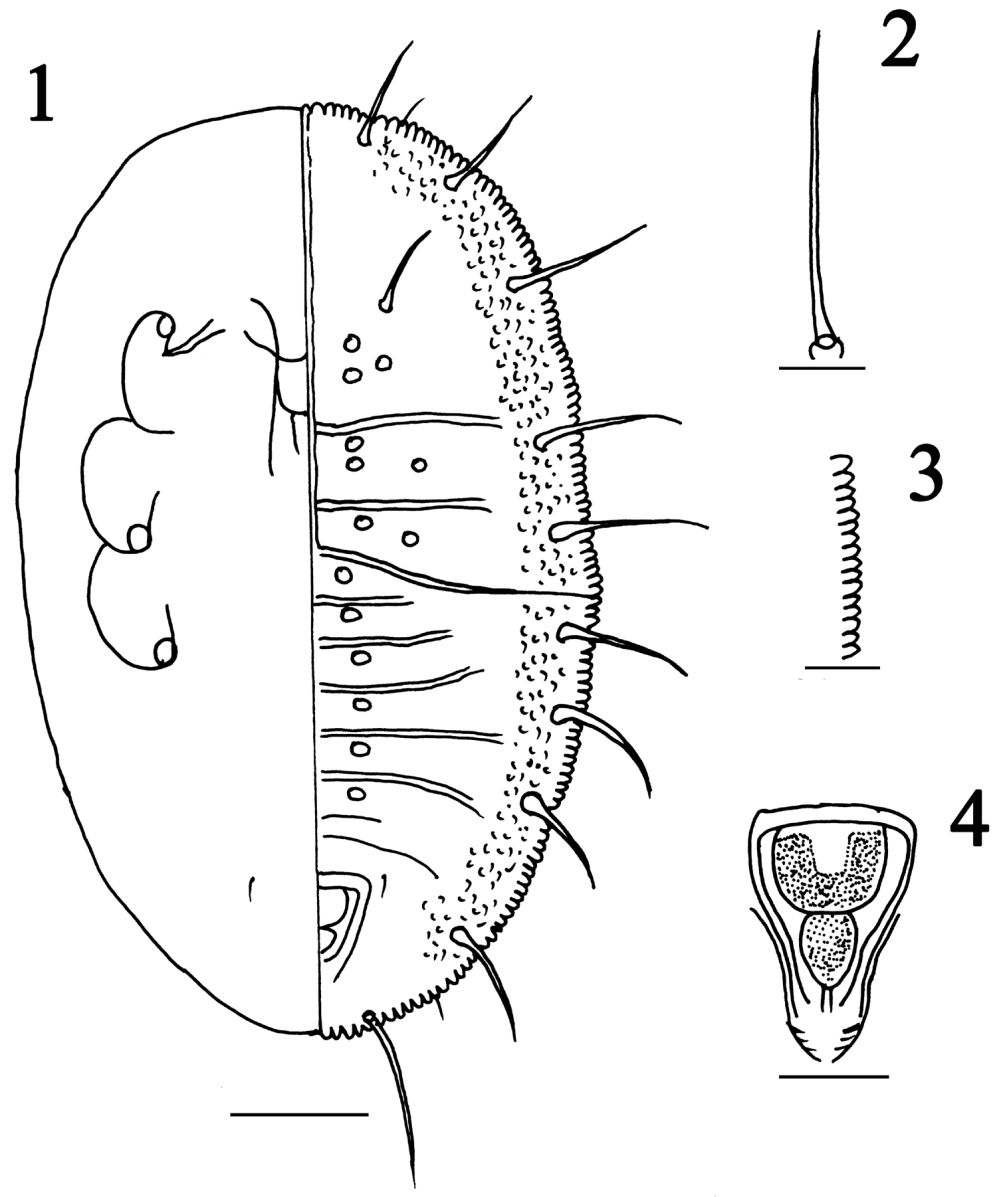
*Metabemisia leguminosa* sp. n., holotype puparium, China (Hainan). **1** puparium, dorsal (right) and ventral (left) views **2** submarginal seta **3** margin **4** vasiform orifice. Scale bars: 0.1 mm (**1**); 0.02 mm (**2**); 0.04 mm (**3**); 0.03 mm (**4**).

**Figures 5–9.**
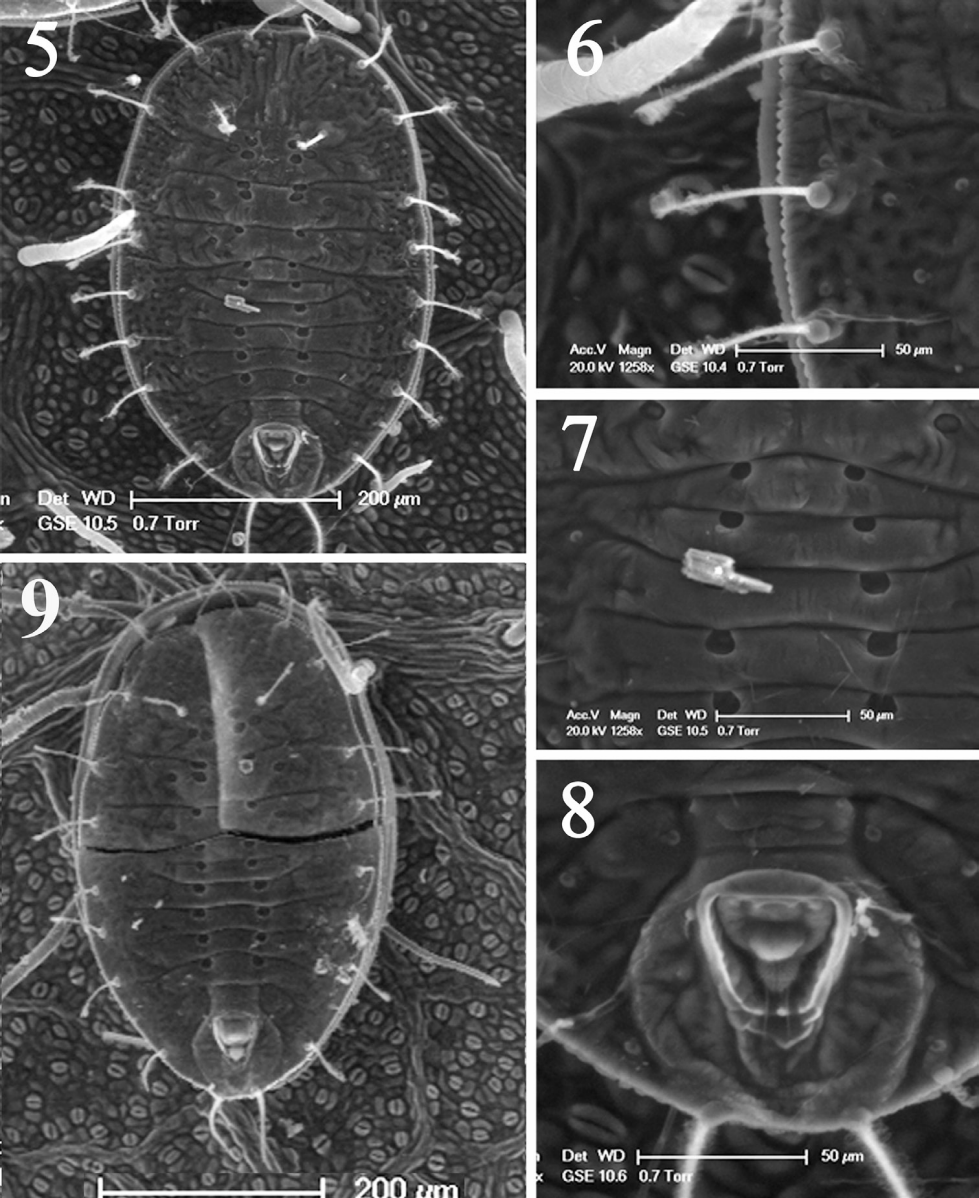
Scanning Electron Microscope (SEM) photographs of *Metabemisia leguminosa* sp. n., China (Hainan) **5** puparium, dorsal view **6** margin and submarginal setae **7** the sub-median depressions on abdominal segments **8** vasiform orifice **9** empty pupal case, dorsal view.

This species is characterized by the submarginal area with ten pairs of subequal longsetae (Figs 2, 6), about 74.6–93.6µm,the presence of 4–5 rows of very small distinct pore along the margin (Figs 1, 5), the absence of the first abdominal setae, and the thoracic tracheal pores being indistinct, the submedian depressions are particularly distinct on abdominal segment I–VI (Fig. 7), vasiform orifice triangular (Figs 4, 8), longer than wide, lingula with a pair of apical setae (Figs 4, 8).

#### Description.

**Puparia (fourth instar).** Body yellowish, elliptical, 581–723 µm long, 306–395 µm wide, broadest at the metathoracic region. Margin crenulate (Figs 3, 6), 23–25 crenulations in 0.1 mm. Approximately 4–5 rows of very small distinct papillae present along the margin. Paired anterior and posterior marginal setae 19–24 µm and 18–22 µm long, respectively.

Dorsum. Submarginal area with ten pairs of long setae, nine of which are subequal in length, about 72.3–76.8 µm, each arising from a small tubercle; caudal setae 90.6–95.4 µm; cephalic setae 41.7–44.8 µm; eighth abdominal setae 8.1–8.8 µm long, first abdominal setae absent. Longitudinal and transverse molting sutures all reaching the margin. A pair of sub-median depressions present on each thoracic and abdominal segment I–VI, approximately 43.3 µm apart. Abdominal segments I–VI nearly equal in length, while abdominal segment VII only about half of abdominal segment VIII, less than half as long as abdominal segment VI.

Vasiform orifice. Triangular, distinctly longer than wide, 62.1–64.6 µm long, 42.6–45.2 µm wide; operculum inverted trapezoid, covering nearly half the orifice, 25.8–29.1 µm long, 32.1–35.2 µm wide. Lingula exposed, knobbed, expand at the base, 13.1–16.2 µm long, 14.2–17.8 µm wide, nearly reaching the hind margin of the orifice, with a pair of apical setae, 10.8–13.1 µm in length. Caudal furrow distinct.

Venter. Thoracic tracheal folds and pores not discernible. Ventral abdominal setae placed on either side of anterior angles of vasiform orifice, finely pointed and 7–9 µm long, 67 µm apart. Adhesive pads present at apex of legs.

**Figures 10–11.**
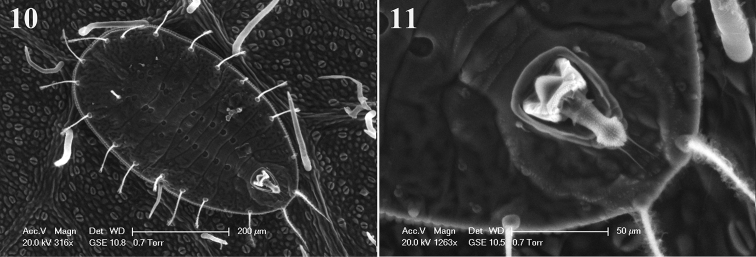
Scanning Electron Microscope (SEM) photographs of *Metabemisia leguminosa* sp. n., China (Hainan) **10** third instar, dorsal view **11** vasiform orifice of third instar.

**Third instar** (Figs 10–11). yellowish, elliptical, about 514–558µm long, 289–303µm wide, the other morphological characteristics are basically identical with the puparia except the vasiform orifice region. The operculum (Fig. 11) protruded in the central part, about 18.6–20.3 µm long, 34.9–36.7 µm wide, and covering about half of the orifice. Lingula (Fig. 11) particularly developed and upward, extending beyond the hind margin of the orifice, about double the length of operculum, 40.7–42.1 µm long; with a pair of apical setae, about 17.4–18.6 µm long.

**Figure 12.**
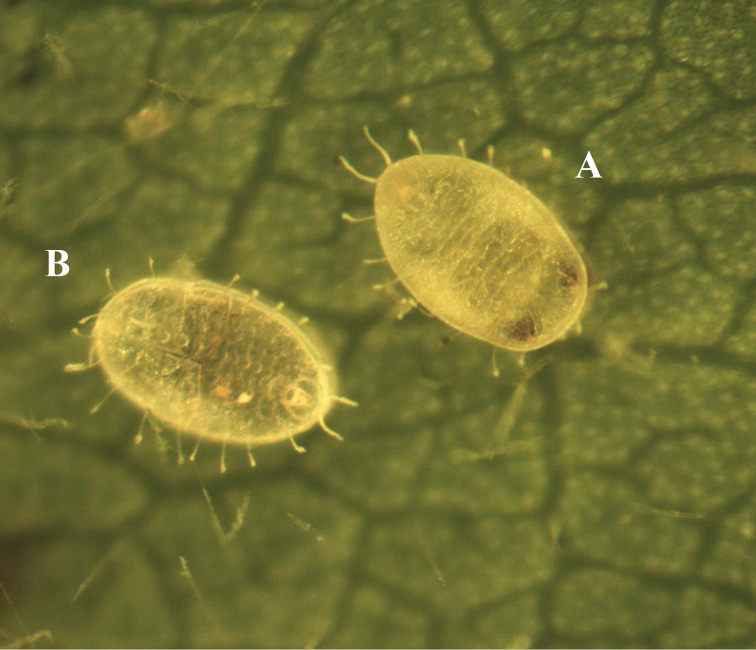
The live images of *Metabemisia leguminosa* sp. n., China (Hainan). **A** puparium, dorsal view **B** empty pupal case, dorsal view.

#### Other instars.

Unknown.

#### Host plants.

Leguminosae.

#### Distribution.

China (Hainan Island).

#### Biology.

Specimens were found in clusters of 5–8 per leaf, centrally on the under surface of leaves. No evident signs of damage have been noted on the host plant. No parasitoids were obtained from the puparia. No ant attendance was observed.

#### Etymology.

The species name was derived from the family name of the host plant; adjective.

### Key to the puparia of *Metabemisia* species

(Puparia characters obtained from original descriptions)

**Table:** 

1	Outline elongate-oval; dorsal bears 14 pairs of submarginal setae; vasiform orifice elongate-cordate; lingular setae absent	***Metabemisia palawana***
–	Outline oval; dorsal bears 10 pairs of submarginal setae; vasiform orifice triangular; lingular setae present	**2**
2	The thoracic tracheal pores distinct; 1^st^ abdominal setae present; without or with only one row of papillae along the margin	**3**
–	The thoracic tracheal pores indistinct; 1^st^ abdominal setae absent; with 4–5 rows of papillae along the margin	***Metabemisia leguminosa* sp. n.**
3	Dorsal without papillaeand ridges; the lingula without basal tubercles, the caudal furrow is longer than the vasiform orifice	***Metabemisia distylii***
–	Margin with 33 pairs of papillae bearing wax glands and with ridges lead from the caudal setae to the vasiform orifice; the lingula tip with basal tubercles weekly developed, the caudal furrow is shorter than the vasiform orifice	***Metabemisia filicis***

### Remarks

The new species resembles *Metabemisia filicis* by the ten pairs of submarginal setae, and by having a pair of sub-median depressions present on the abdominal and thoracic segments. However, in the new species the sub-median depressions are present on abdominal segments I-VI while in *Metabemisia filicis* on abdominal segments I-VII (Mound 1967). In addition the new species differs from *Metabemisia filicis* by the absence of the first abdominal setae, the indistinct thoracic tracheal pores andthe presence of 4-5 rows of very small distinct papillae along the margin. It also resembles the species of *Neomaskellia* Quaintance & Baker, 1913, *Neomaskellia andropogonis* Corbett, 1926 and *Neomaskellia bergii* (Signoret, 1868), but differs from them by the number of submarginal setae and the shape of vasiform orifice (Martin 1987).

## Supplementary Material

XML Treatment for Metabemisia

XML Treatment for Metabemisia leguminosa
